# Women’s health status before and during the COVID-19 pandemic in rural Bangladesh: A prospective longitudinal study

**DOI:** 10.1371/journal.pone.0266141

**Published:** 2022-05-13

**Authors:** Kimiyo Kikuchi, Rafiqul Islam, Mariko Nishikitani, Yoko Sato, Rieko Izukura, Fumihiko Yokota, Nusrat Jahan Khan, Meherun Nessa, Ashir Ahmed, Seiichi Morokuma, Naoki Nakashima

**Affiliations:** 1 Department of Health Sciences, Faculty of Medical Sciences, Kyushu University, Maidashi, Higashi-ku, Fukuoka, Japan; 2 Medical Information Center, Kyushu University Hospital, Maidashi, Higashi-ku, Fukuoka, Japan; 3 Global Communication Center, Grameen Communications, Mirpur, Dhaka, Bangladesh; 4 Division of Integrated Health Sciences, Graduate School of Biomedical and Health Sciences, Hiroshima University, Kasumi, Minami-ku, Hiroshima, Japan; 5 Social Medicine, Department of Basic Medicine, Faculty of Medical Sciences, Kyushu University, Maidashi, Higashi-ku, Fukuoka, Japan; 6 Institute for Asian and Oceanian Studies, Kyushu University, Motooka, Nishi-ku, Fukuoka, Japan; 7 Holy Family Red Crescent Medical College & Hospital, Dhaka, Bangladesh; 8 Department of Information Science and Technology, Faculty of Information Science and Electrical Engineering, Kyushu University, Fukuoka, Japan; UNICEF India, Government Medical College, INDIA

## Abstract

The coronavirus disease (COVID-19) pandemic has widely spread worldwide since 2020. Several countries have imposed lockdown or stay-at-home policies to prevent the infection. Bangladesh experienced a lockdown from March 2020 to May 2020, and internal travel was restricted. Such long and strict confinement may impact women’s health. Herein, we aimed to assess the impact of the COVID-19 pandemic on women’s health by comparing their health status before and during the pandemic. We conducted a prospective longitudinal study in two zones in the Chhaygaon union, rural district Shariatpur, Bangladesh. The study population comprised non-pregnant women aged 15–49 years. We visited the household of all eligible women and invited them for health checkups. The survey staff examined their health status at the checkup camps and conducted questionnaire interviews. In total, 121 non-pregnant women received health checkups both from June 2019 to July 2019 and in October 2020, before and during the COVID-19 pandemic, respectively. Compared with those during the 2019 health checkup, the medians of body mass index, systolic blood pressure, and diastolic blood pressure were significantly higher (22.7 kg/m^2^ to 23.6 kg/m^2^; 110.0 mmHg to 111.0 mmHg; and 73.0 mmHg to 75.0 mmHg, respectively, p<0.05) during the 2020 health checkup. In contrast, urine glucose levels were significantly lower (10.1% to 3.4%, p = 0.021). The lack of physical activity and other inconvenience accumulation caused by the prolonged confinement might have affected their health status. This necessitates local health workers to promote physical activity to prevent health deterioration during the pandemic.

## Introduction

The novel coronavirus (SARS-CoV-2) has spread worldwide and more than 82 million people were infected in 2020 [1]. In 2021, the use of vaccines has gradually decreased the number of cases. However, highly infectious novel strains of coronavirus have emerged in different countries, leading to additional lockdowns or stay-at-home policies.

In addition to the direct impact of coronavirus disease (COVID-19) pandemic on health, it has exerted several indirect impacts. Lockdown and related stay-at-home policies may cause indirect health problems. Previous studies have reported on the impact of social isolation on mental health among children, adolescents, and adults [2, 3]. Additional studies have demonstrated that prolonged periods of isolation at home can limit an individual’s physical activity, leading to health problems such as obesity [4, 5]. People with pre-existing conditions, such as cardiac disease and diabetes, experienced worsening health conditions post-lockdown, such as further increase in already high blood pressure levels and hyperglycemia [6–8].

Approximately 512,000 people were infected with the virus in 2020 in Bangladesh [1], with the first confirmed case recorded on March 8, 2020. Following an increase in the number of cases, the Government of Bangladesh announced a general lockdown on March 26, 2020. The nationwide lockdown was imposed until April 4, 2020, and later extended till the end of May 2020. The lockdown lasted for 2.5 months. Following the lockdown, changes in lifestyle habit and the stay-at-home policy could have influenced people’s health status. However, the impact of COVID-19 on women’s health is unclear.

A previous study in Bangladesh reported increases in women experiencing depressive symptoms and domestic violence (19.9% increase) following COVID-19 lockdown [9]. However, their clinical health status needs further investigation to determine the impact of the pandemic on their health. Thus, we aimed to assess the impact of COVID-19 pandemic on Bangladeshi women’s health by comparing their health status before and during the pandemic in rural Bangladesh.

## Methods

### Study design and site

We conducted a prospective longitudinal study among women who participated in the maternal and child health study, which is a part of the portable health clinic study [10, 11]. The study was conducted in randomly selected two zones out of nine zones of Chhaygaon Union in the district of Shariatpur, Bangladesh. We conducted health checkups for women from June to July 2019 and in October 2020. We also conducted interviews at the health checkups in October 2020 and evaluated their experience during the pandemic.

### Study population and selection criteria

The study population comprised of women aged 15 to 49 years, living in the study site on the day of the health checkup. We only analyzed non-pregnant women and those who attended both health checkups in 2019 and 2020. Pregnant women were not included in this analysis as their hormonal changes may affect the results. Those who refused to participate in the study were excluded. Local health collaborators visited the households of all eligible women in the target area and announced the health checkup dates. We placed checkup camps in the community for three days. All women could visit the camp for a checkup on any of the three days.

### Data collection

At the checkup camps, survey staff collected the health data and conducted the interviews. The survey staff had been trained on the utilization of checkup devices and materials. The following health data were collected: body mass index, blood pressure, blood glucose, urine protein, urine sugar, hemoglobin, pulse rate, body temperature, and peripheral oxygen saturation. We evaluated both blood glucose and urine sugar levels to comprehensively estimate the risk of diabetes. Health data were recorded as raw data and were categorized into four levels, namely: healthy, caution, affected, and emergent according to the criteria in S1 Appendix. Those who were categorized as affected or emergent received medical consultation and a prescription, if necessary. The criteria had been developed by the research team members with medical qualification following their previous experience developing non-communicable diseases criteria [11] and were further reviewed by local physicians. Weighing scales, digital sphygmomanometer, blood glucose test sensor, digital thermometer for women (all above by Omron Corporation, Kyoto, Japan), pulse oximeter (Matsuyoshi & Co., Ltd, Tokyo, Japan), height measurement tape (seca GmbH & Co. KG, Hamburg, Germany), hemoglobin test sensor (Bioptik Technology Inc, Miaoli County, Taiwan), and urine test strips (Terumo Corporation) were used for the checkups.

### Statistical analyses

We descriptively analyzed the collected data for women who received a health checkup in both 2019 and 2020. We compared the raw health data for each checkup content between the aforementioned two periods among all women. In addition, we compared the categorized data, healthy and nonhealthy (caution, affected, emergent) levels, between 2019 and 2020. Furthermore, we stratified the categorized health status according to age group, as it was reported to be related to the health status in a previous study [11]. We then compared the status between 2019 and 2020 through a Wilcoxon signed-rank test or a paired-samples t-test to assess the difference in continuous variables, and the McNemar test to assess binominal difference. The level of significance was set at *p*<0.05. All data analyses were performed using IBM SPSS, version 27.0 (IBM Corp., Armonk, NY, USA).

### Sample size and power analysis

We conducted a post hoc power analysis of 121 samples, based on the overall examined results (1 = healthy, 2 = caution, 3 = affected, and 4 = emergent) between the two periods. We used the SPSS power analysis for paired-samples t-test. The analysis was conducted using several parameters such as the mean (2.07, 2.24), standard deviation (0.550, 0.619) of the overall examined results, Pearson product-moment correlation coefficient (0.436), two-sided test, and type α error (0.05). The calculated power was 0.898. Thus, we considered the sample size sufficient for this study.

### Ethical approval

This study was reviewed and approved by the Institutional Review Board of the Kyushu University, Japan, which serves as the central ethical review board for this study, in response to a written request from the joint research institute, Grameen Communications, Bangladesh for ethical review. Written informed consent were obtained from all participants (or from parents or guardians for minors) prior to data collection. Their participation was voluntary, and confidentiality was maintained. All study procedures were performed following the tenets of the Declaration of Helsinki.

## Results

A total of 217 and 241 non-pregnant women attended the health checkup in 2019 and 2020, respectively. Of these participants, 121 attended in both periods. Their mean age was 29.2 years (SD = 9.4) at the first checkup in 2019. The characteristics of the women who attended both checkups are presented in Table 1.

**Table 1:** Characteristics of women who attended both checkups in 2019 and 2020.

			(n = 121)
Characteristics*		Number	Percentage
Age			
	<20	21	17.4
	21–29	39	32.2
	30–39	44	36.4
	40–49	17	14.0
Housing^†^			
	Building	16	13.9
	Tin-wooden paka^§^	20	17.4
	Tin-wooden kacha^||^	79	68.7
Education^†^			
	Never attended	17	14.8
	Primary school	32	27.8
	Junior school	37	32.2
	High school	17	14.8
	Vocational school	7	6.1
	Bachelor	5	4.3
Occupation^†^			
	Housework	89	77.4
	Student	21	18.3
	Government	2	1.7
	Non-government	1	0.9
	Self-employment	1	0.9
	Other	1	0.9
Marital Status			
	Married	98	81.0
	Single	21	17.4
	Widowed	2	1.7
Number of children^‡^			
	No child	34	30.1
	1	15	13.3
	2	25	22.1
	3	21	18.6
	4≤	18	15.9

* Status at the checkup in 2019

^†^n/a = 6

^‡^ n/a = 8

^§^ Paka: brick and concrete dwelling

^||^Kacha: housing using soil

Table 2 summarizes the health status by comparing the rate of healthy versus nonhealthy (caution, affected, emergent) between 2019 and 2020. The percentages for BMI and systolic blood pressure decreased by 11.6% (CI, 5.9 to 17.3, *p*<0.001) and 7.5% (CI, 2.8 to 12.2, *p* = 0.012), respectively, in the healthy group in 2020. In contrast, the percentages of the healthy group in 2020 increased for body temperature and urine sugar levels by 14.1% (CI, 7.9 to 20.3, *p* = 0.010) and 6.7% (CI, 5.9 to 7.5, *p* = 0.021), respectively.

**Table 2:** Differences in the health status between 2019 and 2020 (McNemar test).

										(n = 121)	
Parameter	Year of checkup	Healthy		Nonhealthy						McNemar p-value^†^	
				Caution		Affected		Emergent			
		n	%	n	%	n	%	n	%		
Overall examined results	2019	13	10.7%	87	71.9%	20	16.5%	1	0.8%	0.629	
	2020	10	8.3%	74	61.2%	35	28.9%	2	1.7%		
Body mass index	2019	89	73.6%	26	21.5%	6	5.0%	0	0.0%	<0.001	**
	2020	75	62.0%	36	29.8%	10	8.3%	0	0.0%		
Waist-hip ratio	2019	56	46.3%	65	53.7%	―	―	―	―	0.719	
	2020	53	43.8%	68	56.2%	―	―	―	―		
Body temperature (Celsius)	2019	91	75.2%	27	22.3%	2	1.7%	0	0.0%	0.010	**
	2020	108	89.3%	9	7.4%	4	3.3%	0	0.0%		
Oxygen saturation	2019	121	100.0%	0	0.0%	0	0.0%	0	0.0%	0.500	
	2020	119	98.3%	1	0.8%	1	0.8%	0	0.0%		
Blood pressure	2019	112	92.6%	5	4.1%	4	3.3%	0	0.0%	0.012	*
(systolic)	2020	103	85.1%	9	7.4%	9	7.4%	0	0.0%		
Blood pressure	2019	104	86.0%	10	8.3%	7	5.8%	0	0.0%	0.189	
(diastolic)	2020	95	78.5%	11	9.1%	13	10.7%	0	0.0%		
Blood glucose	2019	113	93.4%	5	4.1%	2	1.7%	0	0.0%	1.000	
(postprandial)	2020	114	94.2%	3	2.5%	3	2.5%	1	0.8%		
Blood hemoglobin	2019	86	71.1%	33	27.3%	2	1.7%	0	0.0%	0.097	
	2020	98	81.0%	16	13.2%	7	5.8%	0	0.0%		
Pulse rate	2019	99	81.8%	19	15.7%	3	2.5%	―	―	0.678	
	2020	96	79.3%	22	18.2%	3	2.5%	―	―		
Urine sugars	2019	107	88.4%	0	0.0%	12	9.9%	―	―	0.021	*
	2020	115	96.6%	0	0.0%	4	3.4%	―	―		
Urinary protein	2019	107	88.4%	13	10.7%	1	0.8%	―	―	1.000	
	2020	108	89.3%	13	10.7%	0	0.0%	―	―		

*p<0.05

**p<0.01

^†^ McNemar test p-value of "healthy" and "nonhealthy" groups, ^||^ n/a = 2.

We further analyzed the aforementioned significant variables in the non-categorized data (Table 3). In 2020, we observed significant increases in BMI (*p*<0.001), systolic blood pressure (*p* = 0.036), and diastolic blood pressure (*p* = 0.005), compared to the 2019 checkup results. However, the positive rate in urine sugar significantly decreased (*p* = 0.021).

**Table 3:** Change of major biophysical data between 2019 and 2020 (Wilcoxon signed-rank test and paired t-test).

							(n = 121)	
Parameter	Year of checkup		Median	Interquartile range	Number	Percent-age	p-value	
Body mass index^†^	2019		22.7	5.78			<0.001	**
	2020		23.6	6.07				
Blood pressure (mmHg)	2019		110.0	17.5			0.036	*
(systolic)^†^	2020		111.0	21.0				
Blood pressure (mmHg)	2019		73.0	15.0			0.005	**
(diastolic)^‡^	2020		75.0	17.0				
Urine sugar^§^	2019	(-)			107.0	89.9	0.021	*
		(+)			12.0	10.1		
	2020	(-)			115.0	96.6		
		(+)			4.0	3.4		

*p<0.05

**p<0.01

^†^Wilcoxon signed-rank test:

^‡^Paired t-test

^§^ McNemar test, n/a = 2.

**Fig 1:**
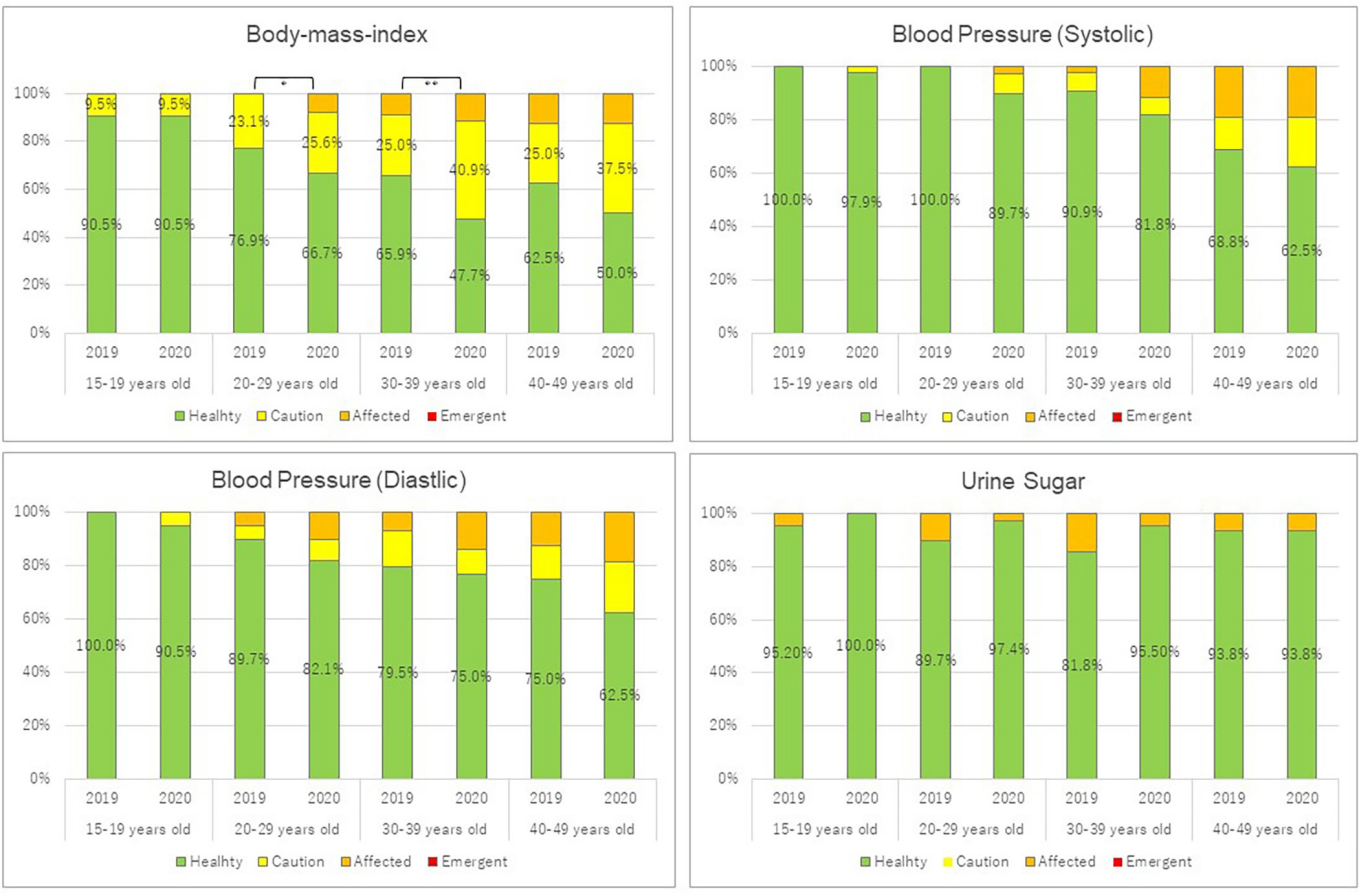
Differences in health status between 2019 and 2020 among the stratified age group participants.

Fig 1 depicts the differences in the health status between 2019 and 2020 with age group stratification. We observed a significant difference in BMI among women aged 20–29 (*p* = 0.035) and 30–39 years (*p* = 0.003). Despite differences in the overall participants, no significant difference was noted in urine sugar levels and blood pressure among the age-stratified groups.

## Discussion

BMI significantly worsened during the pandemic in both continuous and categorized variable comparisons. Similar results were reported in previous studies conducted in Iraqi Kurdistan, Italy, and Zimbabwe with self-measured BMI during the pandemic [12–15]. During the lockdown, researchers reported changes in eating patterns and behaviors as a result of prolonged time at home, which often led to increases in BMI [13, 16]. Some studies reported an increase in high energy food consumption [16], snacking frequency, and enrichment of meals (dessert/sweets consumption at lunch) [13]. These particular tendencies were more significant among women [16]. The aforementioned changes in eating patterns and behaviors might be attributed to emotional and uncontrolled eating. In addition to that, the movement restrictions from the lockdown disrupted the food supply chain, causing the price of vegetables to increase in Bangladesh [17]. In particular, the households that depended on agricultural farming, like our study participants’, faced more food insecurity than those working in the public sector [18], consequently, this affected the people’ s nutrition. Staying at home for longer time periods, health-related anxiety, depression, quality of personal relationships, quality of life, and food insecurity may trigger eating episodes [19].

Lower levels of physical activity and increased time being stationary because of confinement might also increase BMI [20, 21]. We observed nonhealthy BMIs among relatively younger women, aged 20–39 years. The abovementioned age group is usually physically active, thus longer stay-at-home might have significantly influenced their BMI.

Blood pressure significantly worsened during the pandemic in both continuous and categorized variable comparisons. Similar results were reported in Argentina regarding emergency room patients regardless of whether they had severe acute respiratory syndrome coronavirus 2 (SARS-CoV-2) infection or not [22].

According to a systematic review, prolonged stay-at-home, fear of infection, inadequate health-related supplies and information, and poor finances during the pandemic resulted in the development of psychological stressors [23] and they can be a cause of change in blood pressure. In addition, deteriorated BMI levels may also cause high blood pressure. According to studies conducted in South Asia, a higher BMI induced by a lack of physical activity and prolonged sitting time might increase blood pressure [24–26]. In contrast, the pandemic exerted a positive impact on the blood pressure [24]. Further studies are needed to understand the mechanism.

Urine sugar levels significantly improved during the pandemic. Studies conducted in India and the Netherlands reported worsening glycemia during the COVID-19 pandemic in diabetics [7]. Weight gain, decreased exercise, and stress were identified as contributing factors. Our study participants were expected to report increased urine sugar levels under similar conditions, considering their deteriorated BMI and increase in high blood pressure. However, we observed contrasting results, indicating other positive factors might have affected our findings. Adults with type 1 diabetes experienced significantly improved continuous glucose monitoring parameters post-lockdown compared to pre-lockdown values, in an Italian study [27, 28]. The aforementioned study suggests that these positive results were due to a more regular lifestyle during the lockdown, and by spending prolonged time at home, timing and composition of meals improved.

Moreover, the influence of seasonal diet changes, particularly in Bangladesh, might have affected our study’s improved urine sugar levels. Dietary diversity and fruit intake in Bangladesh are the highest during the monsoon season (June–August) [29]. The first health checkup was conducted in the monsoon season in 2019, during which people were out of the *monga* season (poverty and hunger season) and consumed a variety of fruits (such as mango, jackfruit, and lychee). The second checkup was conducted in late autumn in 2020, during which the dietary diversity was similar to the first checkup conditions. However, pandemic-related confinement might have resulted in the restriction of available food and affected food patterns, thereby preventing an increase in glucose level. Despite differences in urine sugar levels, no variation was noted in blood glucose levels. Thus, food consumed several hours before the health check might have affected the urine sugar levels. We cannot make a sufficient judgment from this finding alone; therefore, careful observation of evidence from further research is needed.

Our study had some potential limitations. Considering the public health policies enacted in Bangladesh, we were unable to conduct health checkups in 2019 and 2020 during similar seasons. Seasonal weather and/or seasonal food intake might have affected the examined data. By collecting the data of a similar period in 2021, we may understand more realistic changes that suppressed the seasonal influence. Furthermore, we could not measure the fasting blood glucose level, as this measurement would impede timely health checks, given the large number of women being tested. We were obliged to measure postprandial blood glucose levels, which could have been affected by the meal taken before the test. The available data on socio-economic variables were few in our study and serve as a limitation. If we had collected more confounding factor variables such as household income, household wealth level, husband’s education, and mental health, we could have analyzed more determinants of each health status. However, we also tested the urinary glucose level, which allowed us to estimate comprehensively the diabetic risk together with the postprandial blood glucose. Despite these limitations, the study has strengths. The recruitment of all eligible female residents from the selected study wards supports the generalization of our findings. In addition, similar individuals were longitudinally compared in repeated time points, which enabled demonstrating the impact of COVID-19 pandemic. A further long-term study is expected, which will show the possible changes in the subjects’ health status after COVID-19.

## Conclusions

The COVID-19 pandemic changed our lives and had a significant influence on our health. Our findings revealed changes in women’s health status during the COVID-19 pandemic in rural Bangladesh. BMI and blood pressure increased following lockdown-mediated confinement. The lack of physical activity and related inconvenience accumulation caused by the prolonged confinement might have affected their health status. These findings show that local health workers need to promote physical activity to prevent health deterioration during the pandemic. COVID-19 continuously mutates and results in infection, potentially forcing repeated lockdowns. In anticipation of changes in health status by confinement, a system that enables provision of regular health checkup even during a lockdown period needs to be set up immediately.

## Supporting information

S1 Appendix Portable health system data validation range (extract). (JPG)

S1 Data (PDF)

S1 File (PDF)
