# Supplementary figures and images for: Women’s health status before and during the COVID-19 pandemic in rural Bangladesh: A prospective longitudinal study
Source: PLoS One. 2022 May 13;17(5):e0266141. doi: 10.1371/journal.pone.0266141 (PMC9106176; doi:10.1371/journal.pone.0266141)

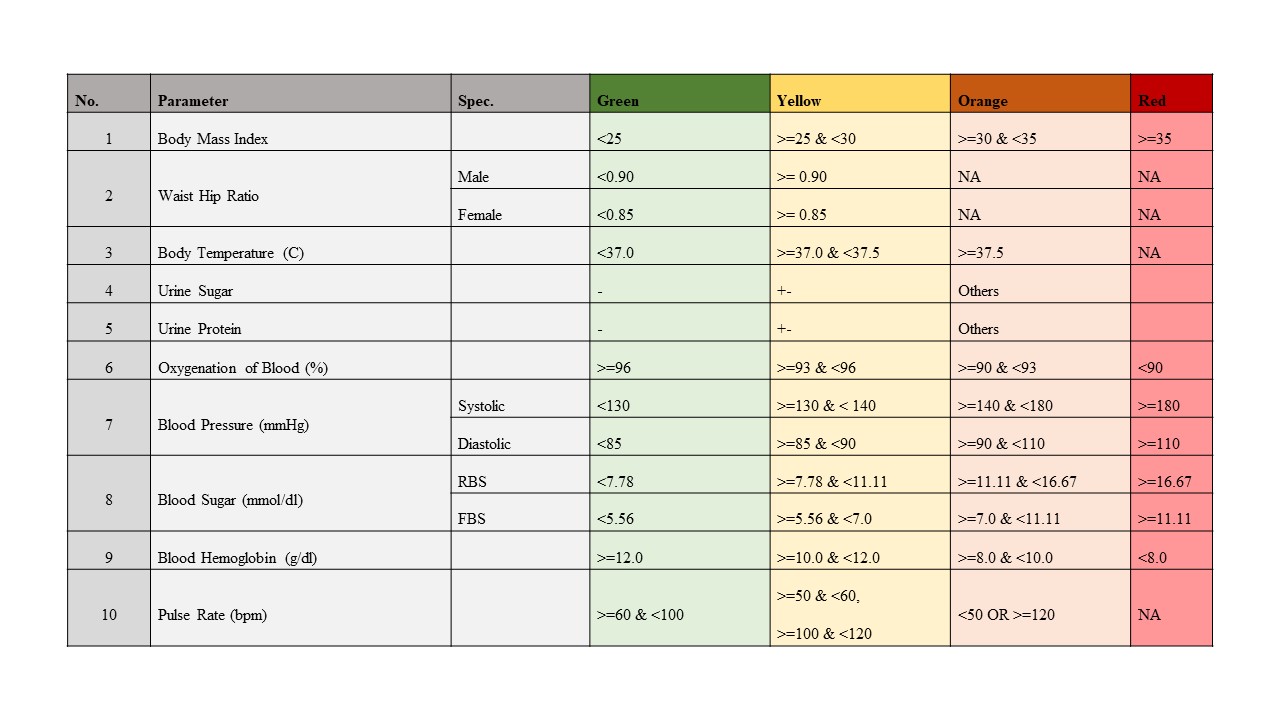

Supplement: S1 Appendix — (JPG) [file pone.0266141.s001.jpg]
