# Supplementary material for: Women’s health status before and during the COVID-19 pandemic in rural Bangladesh: A prospective longitudinal study
Source: PLoS One. 2022 May 13;17(5):e0266141. doi: 10.1371/journal.pone.0266141 (PMC9106176; doi:10.1371/journal.pone.0266141)
Supplement: S1 Data — (PDF) [file pone.0266141.s002.pdf]

age_onche	living_statu	education	education_	occupatior	marital_sta	ever_given	height	weight	bmi_bs	bmi_mid	temperature_bs	temperature_mid	oxygen_of_blood_bs	oxygen_of_blood_mid	bp_sys_bs	bp_sys_mid	bp_dia_bs
31	3	3	1	9	1	1	152.5	56.0	24.1	24.9	99.14	97.52	99	99.00	118	103.00	74
47	3	7	1	7	2	2	161.0	63.7	24.6	25.9	97.77	95.72	98	99.00	97	125.00	75
31	3	7	1	7	2	2	150.0	56.8	25.2	26.9	96.51	98.06	98	99.00	105	130.00	62
30	3	7	1	7	2	2	153.0	67.4	28.8	28.7	97.70	97.52	98	99.00	101	127.00	68
31	3	5	1	7	2	2	158.0	48.7	19.5	22.4	92.21	96.44	98	99.00	111	109.00	62
27	2	5	1	7	2	1	151.5	55.4	24.1	25.2	98.42	97.70	99	99.00	128	125.00	86
24	2	4	1	7	2	2	151.0	46.5	20.4	24.2	98.76	96.80	98	99.00	89	91.00	55
20	3	4	1	7	2	1	157.0	63.2	25.6	17.6	98.11	97.52	98	98.00	106	109.00	71
27	3	4	1	7	2	2	146.0	48.4	22.7	25.2	99.14	98.24	98	99.00	106	111.00	69
38	3	4	1	7	2	2	146.0	63.2	29.7	29.0	94.39	95.90	99	99.00	114	111.00	79
30	1	4	1	7	2	2	147.0	53.5	24.8	25.5	96.62	96.08	99	98.00	86	103.00	53
21	3	4	1	7	2	1	132.0	38.1	21.9	15.0	92.48	96.98	99	99.00	93	98.00	65
19	3	4	1	7	2	2	159.0	64.6	25.6	27.5	98.56	97.16	99	97.00	114	110.00	75
20	3	4	1	7	2	2	146.0	47.3	22.2	18.5	92.59	98.24	98	99.00	102	98.00	73
29	3	4	1	7	2	2	150.0	65.1	28.9	30.0	100.94	97.52	99	99.00	112	132.00	83
22	1	4	1	7	2	2	159.5	75.9	29.8	32.6	99.32	96.80	99	99.00	106	119.00	79
20	1	4	1	7	2	1	150.5	42.3	18.7	19.8	98.46	97.70	98	99.00	114	115.00	65
24	2	4	1	7	2	1	158.5	59.8	23.8	24.7	97.70	96.62	98	99.00	113	89.00	77
47	1	3	1	7	4	2	159.0	59.5	23.5	24.8	97.63	97.34	99	98.00	168	155.00	98
29	3	3	1	7	2	2	151.0	53.1	23.3	26.6	98.60	94.46	99	99.00	119	119.00	75
17	3	3	1	7	2	1	143.0	40.7	19.9	20.2	98.24	97.16	99	99.00	98	109.00	70
30	2	3	1	7	2	2	153.0	42.9	18.3	19.0	97.70	97.16	99	99.00	129	124.00	84
24	2	3	1	7	2	2	149.0	46.4	20.9	20.9	97.48	98.24	99	98.00	117	135.00	76
27	3	3	1	7	2	1	145.0	51.9	24.7	26.6	99.14	98.42	99	98.00	119	93.00	70
25	3	3	1	7	2	2	145.0	62.0	29.5	27.1	98.96	96.98	97	99.00	106	110.00	77
26	2	3	1	7	2	2	145.5	36.5	17.2	21.0	98.46	96.26	97	99.00	100	119.00	62
32	3	3	1	7	2	2	148.0	70.0	32.0	32.3	98.82	96.62	97	98.00	126	115.00	88
30	1	3	1	7	2	2	159.0	60.1	23.8	25.0	98.69	97.34	97	99.00	85	93.00	60
26	3	3	1	7	2	2	160.0	60.4	23.6	25.6	98.56	98.42	98	99.00	99	106.00	61
20	3	3	1	7	2	2	164.0	57.6	21.4	22.5	94.28	96.26	98	98.00	94	96.00	56
39	2	3	1	7	2	2	150.0	39.1	17.4	25.2	97.70	96.08	98	99.00	113	156.00	74
20	2	3	1	7	2	2	153.0	41.3	17.6	18.0	96.03	96.44	99	99.00	101	98.00	62
33	1	3	1	7	2	2	147.5	50.0	23.0	20.7	98.78	96.98	98	99.00	104	105.00	76
20	3	3	1	7	2	2	154.0	45.5	19.2	20.5	95.72	96.80	99	99.00	110	116.00	62
24	3	3	1	7	2	1	138.0	34.1	17.9	23.5	97.34	95.54	99	99.00	94	126.00	59
30	3	3	1	7	2	2	146.0	49.6	23.3	20.8	96.85	98.24	98	98.00	111	111.00	71
30	2	3	1	7	2	2	153.5	50.8	21.6	23.9	97.34	95.90	99	99.00	100	99.00	62
35	3	3	1	7	2	2	143.0	44.4	21.7	26.9	93.97	98.06	98	99.00	115	105.00	77
45	2	3	1	7	2	1	154.0	58.7	24.8	25.3	99.28	98.06	98	99.00	134	135.00	84
22	3	3	1	7	2	2	154.0	61.2	25.8	27.9	97.52	98.96	99	99.00	102	119.00	76
21	3	3	1	7	2	2	151.0	53.8	23.6	23.9	96.08	98.24	98	99.00	111	105.00	74
31	3	3	1	7	2	2	146.0	60.0	28.2	28.8	99.14	99.14	98	99.00	114	102.00	75
26	3	3	1	7	2	2	154.0	52.6	22.2	24.2	99.14	98.42	99	99.00	115	126.00	75
32	1	3	1	7	2	1	147.0	55.9	25.9	25.4	97.88	98.24	98	99.00	120	121.00	81
49	2	3	1	7	2	2	147.0	69.5	32.2	32.5	97.25	97.70	98	95.00	105	111.00	73
23	1	3	1	7	2	1	147.4	39.1	18.0	20.4	91.58	97.34	98	99.00	107	93.00	65
38	1	3	1	7	2	2	159.0	70.4	27.9	29.0	98.06	98.06	99	99.00	128	141.00	89
36	2	3	1	7	2	2	163.5	73.8	27.6	28.1	96.87	98.06	99	99.00	122	121.00	89
25	3	2	0	7	2	2	147.0	64.1	29.7	31.0	97.97	97.16	98	99.00	123	143.00	93
41	3	2	0	7	2	2	153.5	50.3	21.4	22.5	96.04	98.24	98	98.00	96	98.00	66
37	1	2	0	7	2	2	154.5	70.0	29.3	29.8	99.61	96.98	96	97.00	132	135.00	90
34	3	2	0	7	2	2	153.0	50.3	21.5	25.1	98.71	96.98	98	99.00	118	109.00	78
36	3	2	0	7	2	2	149.0	55.2	24.9	29.5	94.60	97.88	98	99.00	117	107.00	82
31	3	2	0	7	2	2	155.0	66.9	27.9	30.8	97.88	98.78	99	99.00	122	112.00	82
18	2	2	0	7	2	1	158.0	60.0	24.0	21.9	99.27	99.50	99	97.00	112	116.00	67
37	3	2	0	7	2	2	149.0	46.8	21.1	20.9	97.48	97.16	98	99.00	102	103.00	72
21	3	2	0	7	2	2	161.0	53.4	20.6	22.3	99.32	96.98	97	99.00	107	104.00	88
33	3	2	0	7	2	2	153.0	57.0	24.4	25.6	97.75	96.44	98	99.00	91	109.00	58
24	3	2	0	7	2	2	150.0	55.5	24.7	19.8	98.73	96.08	98	99.00	115	105.00	84
32	3	2	0	7	2	2	145.0	62.5	29.7	29.2	97.52	97.34	98	98.00	140	122.00	85
30	3	2	0	7	2	2	153.0	60.1	25.7	25.3	98.60	97.88	98	99.00	138	132.00	85
19	3	2	0	7	2	1	141.0	33.7	17.0	18.2	94.10	95.90	99	99.00	98	100.00	67
36	3	2	0	7	2	2	148.0	47.1	21.5	22.7	97.70	95.90	99	98.00	112	111.00	74
37	3	2	0	7	2	2	151.0	43.0	18.9	20.0	98.76	99.32	99	99.00	100	115.00	66
37	3	2	0	7	2	2	148.0	46.7	21.3	23.7	98.42	100.22	99	98.00	117	119.00	75
25	3	2	0	7	2	2	152.0	49.8	21.6	21.6	96.03	96.08	99	99.00	90	120.00	59
24	3	2	0	7	2	2	154.0	53.9	22.7	25.7	98.60	99.86	99	99.00	120	101.00	68
32	3	2	0	7	2	2	149.0	67.4	30.4	31.9	97.52	98.42	99	89.00	105	106.00	72
39	3	2	0	7	2	2	142.0	62.1	30.8	32.9	96.62	96.44	98	99.00	104	140.00	52
30	3	2	0	7	2	2	154.0	43.6	18.4	23.4	97.68	96.44	99	99.00	94	111.00	62
25	3	2	0	7	2	2	150.5	45.8	20.2	20.2	96.08	98.06	98	99.00	97	91.00	61
40	3	2	0	7	2	2	156.5	39.5	16.1	16.5	96.26	96.62	99	99.00	86	100.00	63
15	3	2	0	7	1	1	152.0	44.8	19.4	22.3	95.27	96.44	98	98.00	109	98.00	74
39	3	1	0	7	4	2	146.0	41.7	19.6	21.2	98.60	96.44	98	96.00	111	122.00	73
38	3	1	0	7	2	2	155.0	47.7	19.9	19.5	97.81	99.50	96	99.00	86	110.00	68
34	3	1	0	7	2	2	147.0	53.9	24.9	23.9	94.82	98.06	98	99.00	113	82.00	79
45	3	1	0	7	2	2	153.0	61.0	26.1	28.5	97.54	98.24	97	99.00	134	137.00	74
32	3	1	0	7	2	2	148.5	51.0	23.1	23.9	97.70	94.82	99	98.00	94	119.00	60
38	3	1	0	7	2	2	161.0	81.6	31.5	32.7	98.11	97.70	99	99.00	110	114.00	86
28	3	1	0	7	2	2	150.0	58.2	25.9	17.2	95.95	95.00	98	99.00	81	89.00	59
40	1	1	0	7	2	2	145.0	38.3	18.2	19.5	95.72	97.52	98	99.00	155	177.00	90
41	3	1	0	7	2	2	150.0	43.8	19.5	19.4	97.54	97.88	97	99.00	115	126.00	72
49	3	1	0	7	2	2	147.5	47.9	22.0	22.1	96.08	96.98	98	99.00	108	112.00	67
38	1	1	0	7	2	2	155.5	59.9	24.8	24.4	98.56	96.08	98	98.00	118	101.00	90
26	3	1	0	7	2	1	149.0	54.4	24.5	24.5	99.14	98.06	99	99.00	108	130.00	79
37	3	1	0	7	2	2	150.5	40.5	17.9	19.3	98.55	96.98	97	99.00	85	101.00	62
33	3	1	0	7	2	2	150.0	43.9	19.5	18.2	92.43	95.36	98	99.00	104	113.00	62
31	3	1	0	7	2	2	154.0	53.8	22.7	21.3	97.88	96.08	98	99.00	102	95.00	62
45	2	1	0	7	2	2	149.5	62.6	28.0	27.8	98.62	96.98	98	99.00	108	117.00	82
45	1	1	0	7	2	2	146.0	43.2	20.3	21.3	95.50	96.98	98	99.00	118	112.00	77
26	3	5	1	6	2	1	155.0	56.6	23.6	22.9	98.46	97.52	98	99.00	110	106.00	66
22	3	5	1	6	2	1	154.0	68.0	28.7	28.8	99.32	97.52	98	99.00	120	122.00	90
19	3	5	1	6	1	1	157.5	59.8	24.1	23.5	98.42	98.42	98	98.00	109	113.00	83
22	2	5	1	6	1	1	155.0	50.5	21.0	21.4	99.14	96.80	98	99.00	95	100.00	64
24	2	5	1	6	1	1	147.0	38.7	17.9	18.7	95.81	95.54	98	99.00	98	99.00	56
26	3	4	1	6	2	1	159.0	72.3	28.6	26.6	98.28	98.42	98	99.00	114	113.00	72
20	3	4	1	6	1	1	146.5	45.2	21.1	21.9	95.00	99.14	98	98.00	103	86.00	60
17	3	4	1	6	1	1	144.5	42.0	20.1	22.3	97.34	99.14	96	99.00	102	118.00	67
17	3	4	1	6	1	1	154.0	49.0	20.7	22.5	97.50	97.34	98	96.00	93	99.00	57
17	2	3	1	6	1	1	142.5	38.4	18.9	18.0	97.16	97.34	98	99.00	109	86.00	67
18	3	3	1	6	1	1	147.0	50.0	23.1	23.4	97.48	98.24	99	99.00	128	126.00	82
17	3	3	1	6	1	1	159.0	46.8	18.5	18.2	96.80	96.44	99	98.00	104	89.00	64
16	3	3	1	6	1	1	150.0	39.8	17.7	18.2	99.14	96.80	98	99.00	106	111.00	66
16	2	3	1	6	1	1	154.0	42.7	18.0	18.7	97.52	97.34	98	99.00	97	89.00	58
16	3	2	0	6	1	1	150.0	40.3	17.9	18.2	97.84	96.24	99	96.00	111	95.00	78
15	3	2	0	6	1	1	150.0	59.9	26.6	26.9	97.70	96.26	99	99.00	110	116.00	74
15	1	2	0	6	1	1	139.0	38.1	19.7	20.2	90.23	96.98	99	98.00	100	100.00	66
18	3	2	0	6	1	1	164.0	45.8	17.0	18.6	97.39	95.18	96	99.00	90	83.00	64
16	1	2	0	6	1	1	162.0	58.0	22.1	23.1	97.34	97.52	99	99.00	114	107.00	67
15	3	2	0	6	1	1	153.0	46.3	19.8	20.9	97.70	96.62	98	98.00	98	101.00	58
16	3	2	0	6	1	1	149.0	37.7	17.0	17.3	97.75	96.80	99	99.00	87	78.00	53
30	2	3	1	3	2	2	160.0	56.8	22.2	27.1	99.18	97.88	99	99.00	136	141.00	91
35	2	4	1	2	2	2	161.0	57.8	22.3	23.8	97.63	98.06	99	99.00	123	158.00	78
48	1	7	1	1	2	2	145.0	54.2	25.8	26.4	95.22	99.32	98	99.00	126	136.00	81
41	3	7	1	1	2	2	149.0	61.4	27.7	28.1	96.84	99.14	98	98.00	104	112.00	74
49					2		143.0	62.7	30.7	31.5	98.06	96.80	98	98.00	147	141.00	86
45					2		151.5	56.0	24.4	24.0	97.34	96.62	98	99.00	126	124.00	88
30					2		163.5	62.5	23.4	23.6	94.15	96.62	98	99.00	111	119.00	71
25					2		156.0	53.6	22.0	22.6	95.50	97.70	99	98.00	117	116.00	71
49					2		132.0	37.4	21.5	20.6	95.86	98.60	98	99.00	118	123.00	74
18					1		150.0	41.5	18.4	20.4	98.42	96.44	98	99.00	115	111.00	73

bp_dia_mid	blood_glucose_bs	blood_glicose_mid	blood_hemoglobin_bs	blood_hemoglobin_mid	urinary_glucose	urinary_protein	pulse_rate_bs	pulse_rate_mid
73.00	105	116.00	11.2	12.30	−	−	88	96.00
88.00	85	91.00	12.1	14.20	−	−	88	89.00
72.00	98	110.00	14.4	14.20	+	−	88	95.00
86.00	96	90.00	13.1	14.00	−	−	83	86.00
63.00	104	109.00	10.7	12.50	−	−	75	68.00
82.00	78	98.00	12.5	12.90	−	−	120	122.00
63.00	105	102.00	13.3	13.50	−	+−	100	117.00
78.00	90	76.00	13.8	12.00	−	−	89	77.00
69.00	92	112.00	12.5	13.70	−	−	85	78.00
76.00	82	102.00	14.2	14.10	−	−	75	81.00
67.00	96	91.00	12.0	11.10	−	−	84	84.00
63.00	95	113.00	10.4	14.00	−	−	87	106.00
76.00	128	99.00	14.3	12.60	−	−	104	101.00
70.00	109	93.00	10.8	12.00	−	−	94	100.00
89.00	90	103.00	15.3	14.40	−	+−	122	102.00
80.00	94	148.00	13.4	13.00	−	−	92	93.00
69.00	109	98.00	11.1	12.50	−	−	81	92.00
53.00	87	96.00	13.2	12.10	−	−	116	94.00
94.00	99	124.00	12.4	11.90	−	−	75	89.00
88.00	106	84.00	14.5	14.40	−	−	88	90.00
87.00	96	86.00	12.3	14.20	−	−	76	99.00
86.00	82	98.00	12.4	9.00	−	−	118	105.00
90.00	119	122.00	12.1	8.60	−	−	89	100.00
59.00	109	77.00	12.3	12.00	−	−	83	97.00
80.00	219	225.00	15.5	14.20	−	+−	118	99.00
59.00	96	94.00	11.1	11.30	−	−	113	99.00
75.00	96	102.00	14.8	12.00		−	90	81.00
62.00	136	128.00	13.4	14.00	−	−	80	76.00
63.00	109	98.00	11.8	12.80	−	−	83	84.00
65.00	117	125.00	12.7	8.50	−	−	91	105.00
104.00	94	104.00	12.7	11.90	−	−	93	102.00
65.00	122	105.00	12.3	12.40	−	−	84	81.00
76.00	109	132.00	13.9	14.40	−	−	74	73.00
81.00	96	98.00	11.7	13.30	−	−	83	89.00
74.00	203	308.00	13.2	13.70	+	+−	84	85.00
61.00	80	72.00	11.9	12.40	−	−	71	75.00
54.00	149	125.00	10.5	11.60	−	−	87	76.00
76.00	71	91.00	13.2	12.00	−	+−	90	70.00
84.00	450	265.00	12.0	13.90	+	−	85	82.00
76.00	100	79.00	13.5	11.50	−	−	80	85.00
67.00	103	100.00	12.9	12.40	+	−	108	107.00
64.00	112	113.00	11.1	12.80	−	−	78	65.00
93.00	119	84.00	13.2	12.90	−	−	87	90.00
83.00	97	87.00	12.4	13.00	−	−	92	121.00
82.00	175	112.00	17.3	10.40	−	+−	97	89.00
69.00	107	93.00	12.2	13.30	−	−	95	98.00
92.00	125	137.00	11.9	13.20	−	−	91	85.00
87.00	122	101.00	13.2	13.30	−	−	104	98.00
94.00	134	97.00	12.3	12.90	−	−	99	78.00
61.00	85	92.00	13.9	12.20	−	−	83	67.00
93.00	76	86.00	15.3	9.40	+	−	100	98.00
73.00	81	118.00	11.7	13.80	−	−	78	82.00
66.00	114	173.00	15.7	13.60	−	−	85	89.00
76.00	93	126.00	12.8	12.20	−	−	83	83.00
80.00	127	111.00	14.8	14.40	−	+−	107	119.00
71.00	111	86.00	13.7	12.90	−	−	76	76.00
69.00	89	93.00	11.8	12.50	+	−	82	79.00
67.00	139	82.00	16.0	12.80		−	96	74.00
75.00	107	91.00	11.3	12.80	−	−	110	91.00
81.00	116	110.00	12.4	12.00	−	−	98	86.00
94.00	118	126.00	14.4	13.80	−	+−	98	104.00
68.00	80	90.00	14.7	14.70	−	−	81	100.00
80.00	111	74.00	13.0	12.50	−	−	78	79.00
76.00	94	113.00	11.9	14.10	−	+−	89	95.00
80.00	98	129.00	11.5	13.40	−	−	82	106.00
75.00	106	81.00	12.0	10.00	−	+−	86	66.00
63.00	125	102.00	12.4	11.50	−	−	104	91.00
72.00	151	130.00	13.1	13.30	−	−	104	91.00
88.00	117	95.00	14.1	14.30	−	−	82	94.00
75.00	102	109.00	11.7	13.90	−	−	88	93.00
61.00	101	95.00	13.9	12.60	+	−	88	75.00
68.00	75	96.00	11.4	13.90	−	−	76	99.00
63.00	94	108.00	12.3	13.20	+	−	87	99.00
80.00	100	103.00	12.8	10.90	+	−	81	120.00
75.00	83	95.00	10.9	11.00	−	−	80	70.00
63.00	97	85.00	12.7	13.90	−	−	78	69.00
78.00	100	76.00	11.3	13.20	−	−	69	76.00
78.00	96	72.00	12.8	12.70	−	−	78	72.00
81.00	140	152.00	13.2	11.90	−	−	93	87.00
59.00	69	111.00	11.7	12.50	−	−	85	82.00
95.00	85	115.00	12.6	13.60	−	−	79	75.00
82.00	72	80.00	11.8	12.30	−	−	87	99.00
76.00	101	133.00	12.3	13.10	−	−	76	99.00
73.00	88	87.00	13.3	12.90	+	−	82	79.00
99.00	119	107.00	13.3	13.70	−	−	82	89.00
63.00	103	95.00	11.5	9.90	−	−	75	90.00
60.00	100	89.00	14.3	12.70	+	−	69	72.00
66.00	99	97.00	11.1	12.80	−	−	74	74.00
86.00	88	125.00	12.7	14.60	−	−	76	86.00
70.00	85	112.00	14.1	12.40	−	−	76	73.00
75.00	105	135.00	13.1	12.30	−	−	77	114.00
89.00	93	119.00	11.6	12.60	−	−	121	108.00
63.00	97	94.00	13.3	8.90	−	−	79	94.00
60.00	83	91.00	12.4	11.00	−	−	82	91.00
66.00	77	89.00	12.6	12.60	−	−	74	74.00
64.00	109	103.00	8.2	13.30	−	−	104	81.00
55.00	88	95.00	12.5	13.70	−	−	73	74.00
76.00	129	82.00	13.7	14.20	−	−	118	103.00
69.00	87	82.00	11.8	11.00	−	−	87	98.00
66.00	90	84.00	13.2	9.20	−	−	81	88.00
82.00	95	83.00	12.8	15.30	−	−	105	114.00
63.00	92	86.00	12.0	12.00	−	−	89	91.00
62.00	114	128.00	12.5	14.20	−	−	99	86.00
65.00	86	73.00	14.4	13.80	−	−	76	110.00
68.00	75	93.00	11.9	11.40	−	−	105	113.00
75.00	89	86.00	11.8	11.40	−	−	89	104.00
74.00	131	73.00	15.5	12.10	−	+−	101	104.00
65.00	108	92.00	8.5	12.10	−	−	94	91.00
79.00	95	71.00	13.7	12.70	−	−	88	89.00
61.00	111	85.00	12.7	13.00	−	−	79	83.00
50.00	113	94.00	11.9	12.20	−	+−	77	66.00
103.00	198	267.00	13.9	13.50	+	+	107	93.00
91.00	77	100.00	12.8	12.90	−	−	80	71.00
94.00	112	96.00	11.5	12.60	−	−	89	95.00
77.00	122	118.00	12.3	12.00	−	−	79	93.00
83.00	124	129.00	11.1	12.60	−	−	81	78.00
88.00	98	84.00	13.8	14.10	−	−	84	88.00
78.00	102	78.00	12.6	12.40	−	−	97	98.00
79.00	93	101.00	12.3	12.60	−	−	82	81.00
78.00	114	117.00	15.2	13.70	−	+−	86	96.00
73.00	78	78.00	11.9	12.90	−	−	89	95.00
